# Physicians’ and nurses’ attitudes towards performance-based financial incentives in Burundi: a qualitative study in the province of Gitega

**DOI:** 10.1080/16549716.2017.1270813

**Published:** 2017-01-27

**Authors:** Martin Rudasingwa, Marie Rose Uwizeye

**Affiliations:** ^a^ Institute of Health Economics and Clinical Epidemiology, University Hospital of Cologne, Faculty of Medicine, University of Cologne, Cologne, Germany; ^b^ Company for Research in Social, Behavior and Health, Kigali, Rwanda

**Keywords:** Performance-based financing, incentives, physicians, nurses, attitudes, Burundi

## Abstract

**Background**: Performance-based financing (PBF) was first implemented in Burundi in 2006 as a pilot programme in three provinces and was rolled out nationwide in 2010. PBF is a reform approach to improve the quality, quantity, and equity of health services and aims at achieving universal health coverage. It focuses on how to best motivate health practitioners.

**Objective**: To elicit physicians’ and nurses’ experiences and views on how PBF influenced and helped them in healthcare delivery.

**Methods**: A qualitative cross-sectional study was carried out among frontline health workers such as physicians and nurses. The data was gathered through individual face-to-face, in-depth, semi-structured interviews with 6 physicians and 30 nurses from February to March 2011 in three hospitals in Gitega Province. A simple framework approach and thematic analysis using a combination of manual technique and MAXQDA software guided the analysis of the interview data.

**Results**: Overall, the interviewees felt that the PBF scheme had provided positive motivation to improve the quality of care, mainly in the structures and process of care. The utilization of health services and the relationship between health practitioners and patients also improved. The salary top-ups were recognized as the most significant impetus to increase effort in improving the quality of care. The small and sometimes delayed financial incentives paid to physicians and nurses were criticized. The findings of this study also indicate that the positive interaction between performance-based incentive schemes and other health policies is crucial in achieving comprehensive improvement in healthcare delivery.

**Conclusions**: PBF has the potential to motivate medical staff to improve healthcare provision. The views of medical staff and the context of the area of implementation have to be taken into consideration when designing and implementing PBF schemes.

## Background

In the attempt to improve health services delivery, both developed and developing countries have proposed a wide range of reforms of their health systems. The latest reform efforts have concerned various types of performance incentive schemes such as Pay For Performance (P4P) in developed countries and Performance-Based Financing (PBF) or Results-Based Financing (RBF) in developing countries [1–4]. The focus in the present study is on PBF whereby healthcare providers receive financial rewards for improvements in the quantity, quality, and equity of their services according to pre-defined health targets. The important aim is to motivate healthcare providers and thereby to improve the quality of health services [5–7]. Studies’ results on the effect of PBF on improving health services provide mixed evidence, both in developed countries [3,8,9] and in developing countries [1]. Many studies propose performance-based incentives as a novel strategy that has the potential to stimulate the improvement of healthcare services [3,7,10], particularly in developing countries where the quality of care is still at a low level [2,4]. In developing countries, particularly in Sub-Saharan Africa, the healthcare provision is hampered by numerous constraints, such as ineffective health policies and health systems, weak healthcare management and organization, unmotivated health workers, and inadequate health-worker performance [11–15]. PBF is proposed as a holistic reform approach that aims to improve the aforementioned shortcomings among others in healthcare provision. Whilst there is a general desire to implement PBF in developing countries, there are still several issues that PBF does not substantially address such as the elimination of inequalities in healthcare access by targeting the vulnerable, as well as tailoring the design of those incentive schemes to emergency circumstances (e.g. the Ebola outbreak) and complex healthcare needs [16]. The effects of performance-based schemes on improving the healthcare provision depend on different ‘driving’ factors. These are the size of incentives, the specific contextual factors of health settings, the incentives payment mechanism, the quality indicators included in the incentive programme, the budget and duration of the programme [2,7,17–20], as well as political will and accountability [21]. Often mentioned in the literature is the importance of the context for designing PBF. Hence, incentive schemes need to be responsive to their specific contexts to achieve the strongest improvement in quality [10,18,22,23].

Most studies on PBF have put more focus on quantitative methods to assess the effect of incentives on the improvement of health quality indicators in terms of health services utilization and quality of care. Yet, these studies may not look at the health workers’ perceptions and opinions of performance-based incentive schemes. Taking into consideration health workers’ views and integrating health workers into defining healthcare delivery systems are crucial to achieving successful healthcare provision [24,25]. The available studies on health providers’ attitudes towards performance-based incentives both in developed countries [26–29] and in developing countries [30–33] suggest that health providers generally have positive attitudes towards financial incentives. However, those studies suggest that some health providers were sceptical as to whether performance-based incentive schemes would fairly reward them – they expressed concerns about the PBF’s effectiveness and about possible unintended effects. In Ghana and Burkina Faso, PBF bonuses led to the motivation of health workers who subsequently worked harder and were more committed to service delivery [34,35]. Previous studies have generally focused on the linkage between PBF bonuses and the motivation of health workers without examining the perceptions of health workers about the overall design and implementation process of PBF schemes.

**Figure 1.**
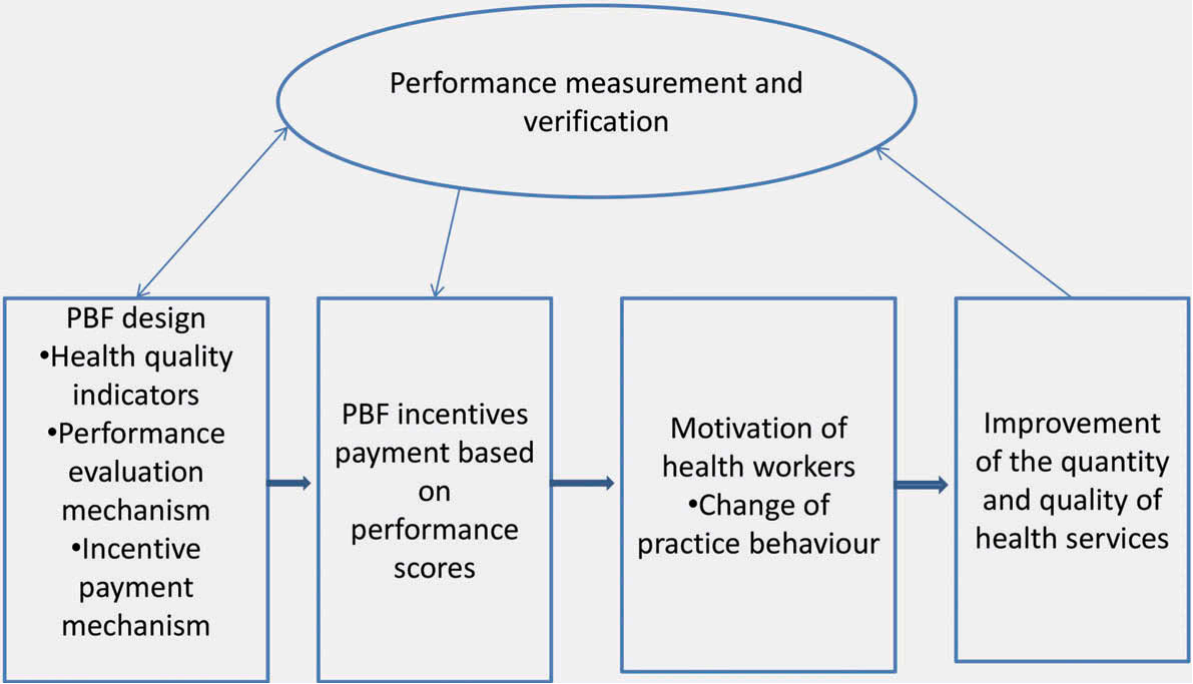
The conceptual framework.

The PBF scheme was implemented in Burundi in 2006. However, still little is known about health workers’ views on how and to what extent that programme helps them to improve the utilization of health services and the quality of healthcare. A study in Burundi about the link between institutional arrangement and health-system performance included healthcare practitioners’ views on the PBF schemes in Bubanza and Ngozi provinces, but at the health facility level, only the directors and administrators were interviewed [36]. The aforementioned study showed that performance-based incentives, accompanied by other factors, encouraged health providers to improve healthcare provision. The current study aims to contribute to closing the gaps of evidence on how medical workers in Burundi view the PBF scheme. The study focuses on frontline practitioners such as the treating physicians and nurses. Another contribution of this study is to assess physicians’ and nurses’ perceptions on the overall design-arrangement of the PBF scheme as summarized in Figure 1.

## Methods

### PBF in Burundi

Like in other Sub-Saharan African countries, the healthcare provision in Burundi is hampered by many challenges, such as low utilization of health services, low motivation of health professionals, low availability of health services, poor quality of care, and ineffective organizational arrangements of healthcare delivery among others [37,38]. In Burundi, health services are provided by health centres and hospitals. Health centres offer a basic package of health services provided by nurses. Hospitals offer a full package of outpatient and inpatient care. There are on average 4 physicians and 30 nurses per hospital, each hospital serving a population of around 150,000 people [39]. The health sector in Burundi was seriously attenuated by a 12-year civil war that ended in 2005.

With the goal of improving the healthcare delivery, the Burundian Government in collaboration with international non-governmental organizations (e.g. HealthNet-TPO [Transcultural Psychosocial Organization] and Cordaid) introduced PBF in 2006 as a pilot programme in three provinces (Bubanza, Cankuzo, and Gitega) and it became a national policy in April 2010. The PBF programme aimed at addressing major challenges in healthcare delivery, such as (1) low utilization of health services, especially for maternal health services; (2) poor quality of care; (3) low motivation of health workers; (4) non-availability of health services around the clock, i.e. 24/7 non-stop service; and (5) poor management and organizational structures of health facilities [37].

The health facilities receive financial bonuses based on the quantity and quality of services provided. The PBF scheme is funded mainly by the Government of Burundi which provides 70% of all the PBF expenses; the rest is paid by its international partners [2]. PBF has become an important financing mechanism to pay healthcare providers. The portion of PBF subsides to the total health expenditure in Burundi has been increasing from year to year, for instance, from 16.5% in 2010 to 46% in 2012 [40]. PBF bonuses accounted for, on average, 20% of the total health facility revenues in 2010 and in 2013 this proportion increased to around 40% [38,41]. This resulted in a fivefold increase of the revenues of health facilities from its first implementation in 2006 up to 2010, translating into an increase of salaries and bonuses for medical staff. For example, the monthly salary (including bonuses) of a nurse increased on average from $75 in 2006 to $262 in 2011 and for physicians from $100 to over $300 in the same period. The bonuses make up 20 and 30% of the total salary of a nurse and of a physician, respectively [42]. The hospital administration uses a pre-defined system to calculate staff bonuses. The bonus calculation formula is called the ‘indices system tool’ and designates the bonuses of each staff member based on qualification, years of experience, and work performance. Although the salaries of health workers are still low compared to the costs of living, PBF bonuses have helped to retain medical staff and to increase qualified nurses in peripheral health facilities [42,43].

Following the manual for PBF implementation in Burundi [38], the PBF scheme is regulated centrally by the Ministry of Health and a provincial committee for evaluation and validation is responsible for the PBF contracts with healthcare providers in each province. The health facilities have the autonomy to allocate the PBF bonuses as they wish. One part of the PBF bonuses is paid as bonuses to medical staff and the remaining money is used for other health facility expenditures, but with emphasis on investments in improvements of the health facility performance.

The healthcare performance in the PBF programme is measured by using quantity and quality indicators. The quantity indicators relate to the numbers of health services provided to patients, such as number of new consultations, number of births attended by qualified staff, and number of HIV and tuberculosis cases. The measurement of quality focuses mainly on structural and process quality indicators as well as some intermediate health outcomes indicators in each health facility: for instance, the providers’ adherence to the national standard of disease treatment; the state and availability of infrastructure, equipment, and materials; the availability and management of drugs; and the patients’ satisfaction [37,39].

The performance payment from quantitative health services is not linked to targets that must be achieved to be eligible for the financial incentives. Rather, the payment is based on the health services provided in the measured period of time. The quantity indicators are evaluated and paid monthly, whereas quality indicators are evaluated and paid quarterly.

Each quantitative health indicator corresponds to a fixed amount of money (e.g. in US dollars – new case of outpatient consultancy: $0.25; inpatient bed day: $0.50; HIV mother treated: $1.00; pregnant woman fully immunized: $0.50; normal delivery by qualified staff: $2.00) and the total payment is the product of the number of cases in that indicator and its unit bonus. The payment of each quantity health indicator is calculated as follows:

(1) 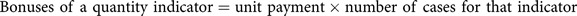

The total payment from the quantity health indicators is the sum of the money earned from all quantitative indicators. The assessment of the quality score of each quality indicator is done using pre-defined composite quality indicators. One composite indicator may contain a certain number of composite criteria, all of which must be met for a provider to be eligible for a financial bonus linked to that indicator. Table 1 shows an example of some composite quality indicators and their respective criteria.

**Table 1.** Maternity quality indicators and their evaluation in hospital.

11. MATERNITY	Available points	Earned points
The delivery room is in a good state Walls are in good state: without fissure and with good painting Pavement without fissure Ceiling in good state Glazed windows with curtains Doors in good state	All criteria met = 10 One criterion not met = 0	…….…. /10
The delivery room is functional Delivery tables are in good state Enough clean water and soap Availability of electric lighting Garbage can with cover Bucket for placenta Baby scale Reanimation table for newborn Obstetric stethoscope Disinfectant Sterile compress Sterile gloves (minimum 10 pairs)	All criteria met = 30 One criterion not met = 0	…….…. /30
Episiotomy materials are available At least 2 boxes of episiotomy materials: episiotomy scissors, surgical forceps, needles, needle holder, tubes catgut and tubes catgut not absorbable	All criteria met = 15 At least one criterion not met = 0	…….…. /15
A ventouse is available and functional At least one physician, one nurse, or one midwife is trained to use the ventouse The ventouse is used (check in patients’ registers)	All criteria met = 15 One criterion not met = 0	…….…. /15
Following instruments and medicines are available for the care of the newborn Sterile cord ligature Sterile umbilical bandage Medical vacuum (bulb dipped in a non-irritating disinfectant or manual or electrical medical vacuum) Warming lamp Tetracycline ophthalmic ointment 1% (used for every newborn)	All criteria met = 20 One criterion not met = 0	…….…. /20
A partogram is available and used At least 10 partograms are in reserve The partogram is filled out once per hour Blood pressure is measured Check 10 partograms	10 10 10	…….…. /30
The partogram provides an active monitoring of the progress in labour: existing of portograph to assisting decision when the curve of monitoring of the labour gets in the action zone. This is available and used Check 10 partograms	20	…….…. /20
The Apgar score is measured and is recorded in the partogram at the 1st, 5th, and 10th minutes	10	…….…. /10
All deliveries are performed by qualified personnel Check this on patients’ records	20	…….…. /20
Adequate waiting room At least four beds and mattresses	10	…….…. /10
The hospitalization room is appropriate and in a good state Beds with matelas of cerecloth without fissure Bed sheet and bedspread at each occupied bed Mosquito net at each occupied bed	All criteria are met = 10 One criterion not met = 0	…….…. /10
Total points	210	…….…. /210

Source: Ministry of Health [38] (Own translation from French).

**Table 2.** Interview guide.

Can you tell us how you were pleased by this PBF programme?
Can you tell us the change this PBF programme has on your work since when it started?
Can you tell us if this PBF programme increases your workload (if it requires you extra time)?
Can you tell us how the quality indicators have been selected? On which criteria have they been selected? ‘Evidence-based therapy recommendations’? Experts’ ideas? Political based?
Can you tell us how the performance evaluation is done and how the PBF bonuses are calculated?
Can you tell us how this PBF programme changed the quality of care provided to patients?
Can you tell us the change this PBF programme has on your relationship with patients?
Can you tell us if you gain money from this PBF programme?
Can you tell us if patients’ care has improved since when this PBF has started? If yes, how? If no, why?
Can you tell us if the quality of care you provide to patients has improved (e.g. diagnosis, treatment, complications, mortality) since when this PBF programme has started? If yes, how? If not, why? More details about structures, process, and outcomes measures.
Can you tell us if in-service trainings of physicians and nurses have improved since when this PBF programme has started?
Can you tell us if the number of patients has increased since when this PBF programme has started?
Can you tell us if the results of laboratory tests are processed in a better way since when this PBF programme has started?
Can you tell us if the collaboration between different hospitals’ services related to treatment of patients has improved since when this PBF programme has started?
Do you have anything else you can tell us regarding this PBF programme since when it started?

At the time of our interview, the quality of health services was assessed using two methods: quality indicators (60%) and a patient satisfaction survey (40%). The first method gives the technical quality of the health facility and the second gives the subjective quality of care received by the patients. Patients are interviewed using random sampling to give their satisfaction on different aspects of health services and at the end a score of the patients’ satisfaction is calculated. During the patient interview, patients are also asked if they got the health services recorded on their treatment records. This serves as a proof of health providers exactly recording the health services they provide to patients. In addition, this serves as a strategy to avoid a situation where health providers can ‘game’ or manipulate the incentives programme by recording health services that they did not provide to patients. The global quality score, which can range between 0 and 100%, is the arithmetic mean between the scores of technical and subjective quality. The quality bonus is rewarded only if a health facility achieves a quality performance of 70% or above. For quality scores between 50 and 70%, there is no payment of quality bonuses. For quality scores below 50%, the health facility gets penalized by losing 25% of the PBF bonuses earned in the previous quarter. The maximum possible quality bonus is 25% of the total bonuses rewarded to the quantitative indicators and is calculated as follows:

(2) 
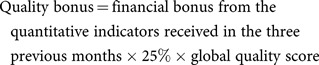

In 2006, the Government of Burundi removed the user fees for institutional deliveries (normal and caesarean childbirths) and healthcare for children under age five at public and faith-based health facilities. From 2006 to 2010, these free services were incorporated into PBF schemes, only in PBF health facilities, then from 2010 onwards, in all public and faith-based health facilities after the scaling up of the PBF scheme nationwide [38].

### Conceptual framework

In PBF, health providers receive incentives for healthcare provided and this aims to motivate them to improve the quality of care [5–7,44]. The healthcare performance is measured based on pre-determined quantity and quality indicators. Based on PBF theory [45] and the PBF scheme design in Burundi [36], as well as inputs from experts in that field, we developed a simple conceptual framework (Figure 1) illustrating the main features of PBF hypothesized to influence healthcare providers in improving the healthcare services.

The conceptual framework describes the PBF design structures in five dimensions:

1. The first step in designing PBF schemes is to define the health quality indicators to incentivize. The performance evaluation and the incentive payment mechanisms have also to be a priori well clarified.
2. The PBF incentives are calculated based on the achieved performance scores.
3. It is hypothesized that PBF incentives motivate the health workers to change and improve their ways (behaviours) of providing healthcare.
4. The ultimate goal of PBF incentives is the improvement of the quality and quantity of health services. It is hypothesized that the motivated health workers will make more efforts to improve the healthcare provision.
5. The performance measurement and verification is an important aspect for the success of PBF schemes. The improvements in the quantity and quality of health services are measured and the PBF incentives are paid to providers based on the obtained performance scores. The performance measurement and verification is a continuous process of verifying if the outputs are in line with what was intended to achieve (targeted results). This helps to redesign or to adjust the PBF scheme if needed.

The conceptual framework guided us in choosing the main themes of our interview and in formulating our interview questions listed in Table 2.

In the Burundian PBF scheme, health facilities get performance incentives every quarter based on their quarterly performance scores in the utilization and quality of health services. At the end of the evaluation, using a standardized evaluation grid (an assessment checklist), each health facility gets an overall performance score, which is then used in a formula to calculate the performance incentives of the quality component. Based on its design, the Burundian PBF creates an incentive for health providers to achieve a high quality performance score and treat a greater number of patients. This study reviews to what extent health workers are motivated and influenced by this scheme.

### Study location and settings

The data collection took place from February to March 2011 in the province of Gitega. The study location was purposely selected based on its experience with the PBF scheme since its first implementation in Burundi in 2006. Gitega Province is one of the three provinces (Bubanza, Cankuzo, and Gitega) that piloted the PBF from 2006 to 2008 [37,46,47]. In addition, the logistical support by HealthNet TPO lent itself more readily to the execution of the study in Gitega Province rather than in the two other provinces. The province of Gitega has five hospitals. Our study was executed in three hospitals that were selected based on their levels of PBF performance scores (high, intermediate, and low performers) at the study time.

### Study design

The cross-sectional study used a qualitative method of face-to-face, semi-structured, and in-depth interviews using ‘open’ and ‘semi-structured’ questions [48]. The conceptual framework (Figure 1) guided the development of the interview guide. The interview guide was tested in another hospital by the first author on 10 physicians and 10 nurses and following the success of the test interview no changes to the content of the guiding questions were necessary. Only some language terminologies were revised.

### Study population and sampling

The sample of the study consisted of 36 health workers encompassing 6 physicians and 30 nurses working in the three hospitals. The interviewees were purposively selected according to their availability, and the 1:5 ratio of physicians to nurses interviewed relates to the scarcity of doctors available in general. The nurses were deliberately selected in different hospital departments until data saturation was reached (no new information emerged). All the physicians who were available at the interview time were interviewed. The senior supervisory staff at the facilities in the form of the medical directors (physicians) and chief nursing-officers (nurses) of the hospitals were also interviewed. In the Burundian context, since there are few physicians, nurses play a big role in healthcare delivery and are in charge of the majority of healthcare services in the hospitals. Ten nurses were interviewed at each of the three hospitals.

### Data collection

The data was gathered by means of interviews and observations at the study settings. The interviews focused on the lessons learnt from the potential effect of the PBF scheme on healthcare provision including the effect on patient reception, patient treatment, quality of healthcare, hygiene, availability of laboratory tests, availability of medicines and materials, and cooperation between the different departments of the hospitals (e.g. internal referral and medical information exchange).

One interview was conducted in French because the interviewee did not understand Kirundi and the others in the Kirundi language. The interviews were conducted and translated into English by the first author. Appropriate ethical permission was obtained from local health facilities’ administration.

### Analysis

The data was analysed using thematic analysis, a methodologically flexible approach for qualitative research [49]. The interview content was analysed by a careful reading and re-reading of the interview transcripts. The codes were developed and then grouped into categories that emerged from similar interviewee views related to the study objectives. These categories were sorted and grouped to generate the themes and subthemes. The main focus was laid on the themes related to the conceptual framework. The categories of interest were analysed using the interviewees’ responses on how and to what extent PBF had helped them to change their way of providing healthcare. The analysis focused on the respondents’ points of view and satisfaction towards PBF in the study health facilities.

## Results

As shown is Table 3, 6 physicians and 30 nurses were interviewed. The average age of the respondents was 38 years (SD: 8.7) for physicians and 31 years (SD: 12.1) for nurses. Gender-wise, the majority of the respondents were women with 3 female physicians and 18 female nurses, whereas the men consisted of 3 male physicians and 12 male nurses.

**Table 3.** Respondents’ characteristics.

	Respondents	Physicians	Nurses	Total
	Number	6	30	36
	Average age	38 (SD: 8.7)	31 (SD: 12.1)	
Men	3	12	15	
Women	3	18	21	

Note: SD: Standard deviation.

The main themes that emerged from the data analysis were (1) the health quality indicators, (2) the performance evaluation mechanism, (3) the financial incentives payment, (4) the motivation of health workers, (5) the quality of health services, and (6) the utilization of health services. The subthemes were incorporated into related main themes. The identified subthemes were: infrastructures, process of care, patient reception and treatment, hygiene, availability of laboratory tests, availability of medicines and materials, cooperation between the hospital departments, knowledge, skills, education, training, health policy and organization, and the number of health workers. The findings of this study show that all the respondents had positive attitudes towards the PBF in helping and motivating them to improve the quantity and quality of health services. They stated that the PBF positively influenced their work. A chief nursing officer stated:

The PBF creates an incentive to increase the attendance rate and the quality of healthcare. We now offer nonstop services to patients 24/24 hours and 7 days/7.

The quality improvements mainly included structures and process quality indicators, but also the relationship between the patients and the health workers. Table 4 illustrates a summary of the respondents’ views showing the extent to which the PBF scheme influenced health workers to improve health services in the study hospitals.

**Table 4.** Summary of healthcare improvements as stated by the respondents.

Healthcare dimensions	Examples of indicators	Very well improved	Well improved	Little or not improved
Infrastructure features and hygiene	Availability and state of facility buildings, beds with mosquito nets, electricity, water, sterilization and decontamination unit, maintenance and repair system, kitchen for patients with all necessary equipment, bathrooms and toilets, general hygiene	✓		
Process features	Adherence to national treatment guidelines: diagnosis, treatment, preventive care	✓		
Health outcomes	Mortality and survival rates, complications, readmission rate, hospital-acquired infections, health-related quality of life			✓
Utilization of health services	Number of patients treated	✓		
Availability of medicines and medical materials	Sufficient essential drugs and medical materials in use and storage, adequate purchasing mechanism		✓	
Physician/nurse–patient relationship	Friendly reception, detailed information on diagnostics, treatment and follow-ups, time for questions and advice, confidentiality and privacy	✓		
Cooperation between hospital departments	Internal referral system (e.g. referral and counter-referral forms), medical record documentation, communication system	✓		

### Health quality indicators

The respondents mentioned that the quality indicators included in that scheme were mostly structure and process quality indicators and some intermediate health outcomes. They stated that those quality indicators were developed based on Burundian treatment guidelines by PBF experts in collaboration with the Ministry of Health. The quality indicators referred to the availability of business plans, financial management systems, materials, equipment, electricity, water, rooms, mosquito nets in inpatient wards, care management, medicines management, hygiene, patient reception, and health quality indicators of some medical indications (maternal and child health services, malaria, tuberculosis, HIV, diarrhoea, surgery, and family planning). Some respondents (n = 14, 38.9%) mentioned that there were other important medical indications that were left out of the scheme that should be included (e.g. dermatology, hepatitis, diabetes, gastroenterology, and heart diseases). The request for the inclusion of new quality indicators was primarily financially motivated. They also complained that the unit bonuses for quantity indicators were small and wished their increase. One nurse stated:

there are other indicators that are not included in the PBF and for them we do not adhere to the norms of the Ministry of Health. And for the included indicators, their unit bonuses are small.

A chief nursing officer stated:

The hospital gets 20,000 Burundian Franc for a caesarean delivery while its costs are estimated at 200,000 Burundian Franc. The mothers do not pay use fees. The services are free for them.

All the interviewees stated that the health services outside of the incentive scheme were not neglected in favour of those which were encouraged by incentives. However, the services under the incentive scheme were afforded more time than those outside the scheme, as they were more carefully documented to maintain comprehensive patient records for facilitating a good performance evaluation. The performance assessment includes only the health services that are under the PBF scheme. One physician stated:

What I can tell you is that this programme has increased our workload for providing better services to all patients than it was before. We spend more time on incentivized services by filling out related patient records because the hospital administration requires us to do so – so that we will not get little bonuses.

Most of the interviewees (n = 32, 88.9%) mentioned that they were not part of the expert team that designed the PBF scheme and were not even consulted. This omission of the inputs and opinions of frontline health practitioners was criticized. Only hospital directors, hospital administrators, and their deputies had participated in the workshops where the experts discussed the design of PBF and the possibilities of its implementation. The interviewees stated that it would have been better to include practising physicians and nurses in the design of that scheme so that they could express their opinions as to how PBF should be designed.

### Performance evaluation mechanism

The respondents stated that the performance evaluation was clearly defined and executed according to a clear evaluation tool (performance scorecard). The performance assessment was executed by an evaluation team using pre-determined quality indicators with corresponding criteria that had to be met in order to attain a performance score. Most of the respondents (n = 30, 83.3%) believed that the performance evaluation was extremely stringent because if one criterion of a quality indicator was not met, the hospital got no performance score for that quality indicator. One physician stated:

The evaluation criteria were not well chosen because if one criterion is missing in a patient record, the evaluators give zero points even if the patient was well treated.

The evaluation method got changed over time without informing the frontline health workers (physicians and nurses). One physician stated:

when the evaluators come, they change the evaluation method from time to time without informing us in advance.

### Financial incentives payment

The interviewees stated that the incentives payment mechanism was comprehensible and clearly defined. The amount of performance-based incentives paid to health facilities was based on the PBF performance score of each health facility. The incentives are not paid directly to hospital staff, but are credited to the hospital accounts. The hospital administrators have the autonomy to allocate the earned incentives as they wish. The hospital directors mentioned that the hospital administration added the performance-based bonuses to the other hospital revenues and then allocated the money in hospital general expenses (e.g. operation costs, purchasing of medicines and medical equipment, fixed staff salaries, different repairs, etc.) and quality improvement projects. The rest of the money (total revenues minus total expenses) was used in paying bonuses to the hospital’s staff. All the respondents stated that they were happy with the bonuses. However, the insufficiency of the incentives was often criticized. In addition, the payment of the incentives seemed to be severely delayed on many occasions. PBF incentives changed over time, based on the hospital’s performance, in such a way that medical staff were not able to estimate the expected performance incentives. Some respondents (n = 16, 44.4%) expressed dissatisfaction at the proportion of the PBF incentives which the hospital administrators allocated to general expenses and quality improvement measures, leaving only a small amount of money as bonuses for hospital staff. In one hospital, most of the respondents (n = 11, 84.6%) lamented that the hospital administration invested such a large part of the money in quality improvement projects that the hospital staff received no performance bonuses for some months. The director of this hospital defended his action with his commitment to improve the quality standards of the hospital. He stated that the investments would attract many patients, that the hospital will make thereby more money in the future, and that hospital staff would then get more bonuses. This director stated this as follows:

through our investments in quality measures, many more patients will come to our hospitals. With many more patients and good quality measures, we will get more PBF incentives and more revenues from patients or their health insurances. All this will be passed down to our hospital staff in the form of more performance bonuses. To achieve these goals, staff will have to accept less performance bonuses in the short-term as an investment to gain greater performance bonuses in the long-term future by our investing in quality improvement measures.

During the interview time of this study, the first author attended a meeting in one of the study hospitals, where the hospital administration had invited the hospital health committee (representatives of health staff from all hospital departments) to explain to them how the bonuses for medical staff were calculated and the reasons why a big part of the money was invested in quality improvement projects. It was noted that the general consensus among the health committee members showed that the majority (seven from nine who attended the meeting) wanted the hospital administration to allocate a lower percentage of the PBF incentives to other expenditures in order to afford medical staff more in bonuses. The majority of the health committee members desired that the PBF incentives should only be allocated to staff bonuses. One member of the hospital health committee stated:

in other health facilities, especially in health centres, we hear that medical staff get big performance bonuses, but in our hospital we get little bonuses because the hospital administration uses the PBF money for other hospital expenditures. It is not fair that our colleagues in other health facilities get more bonuses than what we get when we all work hard to serve patients.

### Health workers’ motivation

All respondents claimed to have been motivated by the financial incentives. They stated that after the PBF implementation, they made additional efforts in healthcare provision because their efforts would be rewarded monetarily. Absenteeism and unjustified breaks were reduced. They stated that they modified their practice behaviour by a closer adherence to national treatment guidelines as was required by the PBF programme. They added that their goal was always to enable the hospital to get the highest possible PBF incentives, which was only achievable by attaining very high performance scores. They pointed out that the PBF scheme has created a spirit of working better and making more effort, and also of changing practice behaviour towards quality improvement. A nurse from one of the hospitals stated: ‘without incentives, there is no motivation to work better for the patients.’

A nurse from another hospital gave her point of view in four short words: ‘No interest, non action.’ A nurse from another hospital stated: ‘financial incentives motivate us to deliver good quality of care.’ However, getting low or no bonuses on some occasions created feelings of dissatisfaction that could dampen the medical staff’s motivation. The respondents wished to get monthly PBF bonuses for being more motivated. One physician stated:

PBF scheme requires a lot of work. But we get little PBF bonuses. Our salaries are lower compared to the costs of life. It is not easy for us. PBF bonuses should be raised and paid monthly.

There were no findings about significant crowding-out of intrinsic motivation. All the respondents acknowledged that the medical profession of saving lives strengthened them not to give up in case of discontent with bonuses. One nurse stated:

we work hard to get PBF bonuses. If we do not get these bonuses, we feel disappointed and somehow discouraged. But we try to treat patients as we should because this is our profession.

### Quality of health services

All the respondents stated that the PBF programme contributed to the improvement of structures and the process of care. The respondents could not however register any remarkable improvement in health outcomes (e.g. readmission rate, late complications, mortality, and health-related quality of life). The main reason given by the respondents was that the treatment knowledge and skills for achieving better clinical outcomes did not improve under the PBF scheme, for instance the in-service training did not improve. One physician stated:

Because of the increase of patients, there is no time for trainings. Only few physicians and nurses go to trainings if this is even possible.

The improvement of the process of care in terms of closely adhering to national treatment guidelines was also backed by an amelioration of the cooperation between hospital departments. The respondents mentioned that PBF fostered cooperation between different departments and led to a better exchange of information, such as patient records being filled out well and well-administered, and the upgrading of the internal patient referral system. The PBF scheme required the providers to complete patient records more comprehensively and accurately. However, because physicians and nurses were few in hospitals, and patients had increased in numbers, the respondents registered the time they spent filling out patients’ records as time lost to patient treatment care. Sometimes, the physicians and nurses ‘gamed’ by reporting services that they did not provide to patients. They suggested that this be carefully considered in organizing healthcare delivery. One physician noted:

This PBF programme is good. If we do all what it requires, patients benefit from it. However, this programme is not applicable in a district hospital because health workers are few. We lose a lot of time by filling out patient records and do not get enough time for patients’ treatment. We have to fill out the patients’ records as required otherwise we do not get PBF incentives. PBF evaluators look at patient records. This leads us sometimes, when we do not have enough time, to fill out patient records at the end of the work and sometimes we report also services that we did not provide to patients.

The general infrastructures, supplies, and equipment of the hospitals were also improved. The director of one of the hospitals stated:

we improved the hospital’s infrastructures, hygiene, and medical equipment with financial incentives income and in the future we want to continue investing more in quality improvement using financial incentives. This incentives programme helps us to improve the quality of care.

The director of another hospital stated: ‘financial incentives help us to improve the structural infrastructure of our hospital.’ One of the nursing directors stated: ‘Financial incentives help us to focus on healthcare quality improvement; this programme will help us to change our mindset and develop a culture of delivering a better quality of care.’ The availability of medicines, medical materials, and laboratory tests slightly improved through PBF in the study health settings. One chief nursing officer stated:

Under PBF, medico-technical and material equipment improved. For example, electric generator, tensiometers, telephones, etc.

The interviewer also made some observations at the hospitals regarding hygiene, facility infrastructure, and equipment and had observed adequate hygiene measures in all hospitals. In the best-performing hospital among the study facilities, a new and well-equipped building for maternal healthcare was observed.

Some challenges in providing high quality of care were mentioned by the respondents. The wider challenge was the general shortage of health workers, especially physicians. The majority of the respondents (n = 34, 94.4%) stated that often the number of patients had increased, and this translated into the few health workers having to work harder to cope with the greater workload. A nurse stated:

the patients have increased but the medical staff are few. […] We also have few laboratory technicians. This is a big problem for us.

All respondents also stated that a high quality of care could only ultimately be achieved when the medical staff’s knowledge and skills in treating patients were sufficient and up-to-date.

The scarcity of adequate core infrastructure (buildings, beds, health information system), lack of good medical equipment and materials, and ineffective drugs procurement and management were also mentioned as potential hindrances to the delivery of a high quality of care.

### Utilization of health services

The majority of the respondents (n = 30, 83.3%) believed that the quality improvements related to the PBF programme contributed to an improvement in the relationship between medical staff and patients, resulting in the increase of health services utilization. However, they mentioned that financial barriers and lack of a comprehensive social health protection hinder many people from accessing health services. The interviewees mentioned that there were remarkable positive changes in the processing of the patients’ reception because this service was incentivized. The patients were offered more time for questions and treatment explanation in a better and friendlier manner compared with before the PBF scheme. The health providers wanted patients to come to their hospitals again so that they could earn more PBF incentives, thus, they took more time making patients feel welcome and encouraged them to come again to the hospital. The improvement of the hospital infrastructure also attracted more patients. The director of one of the study hospitals stated:

we built a modern and well-equipped maternity ward with money from PBF incentives. In addition, PBF has helped us to improve maternal health services, attracting many more women to our hospitals for maternal healthcare.

However, the waiting time to get treatment was not reduced. One physician stated:

The patients have increased but the number of health workers have not increased. Patients have to wait a long time for treatment.

## Discussion

Most of the interviewed physicians and nurses in this qualitative study said that PBF positively influenced delivery of healthcare. It improved the quality of care and increased utilization of health services. This improvement was primarily evident in the areas of structures and process health indicators. Improvements in health outcomes were not witnessed by the respondents. Based on our best knowledge, there is no study on the effect of PBF on health outcomes in Burundi. These findings are in line with other studies that analysed the effects of PBF in Burundi, which also found that PBF mainly stimulated the improvement of structural and process features of healthcare quality and utilization [37,46,47,50]. Burundi, as many other developing countries, faces the challenge that its infrastructure is in an early stage of development. Health workers in many developing countries do not strictly adhere to treatment guidelines, resulting in an insufficient quality of care [51–53]. Such deficits naturally hinder the delivery of good care. The respondents in the current study thought that advances in health service utilization were largely achieved by the improved relationship between medical staff and patients. With the aim of gaining more bonuses from an increased volume of patients, medical staff improved their way of receiving, interacting with, and treating patients. The interviewed physicians and nurses in this study stated that under PBF, some health indicators improved while others did not. The improvement levels also varied (see Table 2). These findings confirm those of previous studies on PBF in Burundi [46,47,50] and in other developing countries [1,22] that indicated different ranges of improvements and non-improvements in health services. Based on the findings of those studies, it can be noted that the success of PBF also depends on the context in which the healthcare is being delivered. Each facility functions according to the availability of health workers and their skills and knowledge. Therefore, the outcomes would be relative to the level to which the healthcare infrastructure was developed in each setting, including available medical equipment and efficiency of the administration and general organization of the facility. As previously stated, the respondents attested to giving more effort to indicators under the incentive scheme, although it was confirmed by respondents that non-incentivized services were not left deficient because of time spent on incentive-based tasks.

The involvement of healthcare providers in all steps of implementing PBF is crucial. To ensure the success of a health policy, Edwards recommended that medical personnel, with their knowledge and experiences, should be integrated into the design and implementation of the scheme. Thus, this involvement, earlier on in the process, should inform the whole project and hopefully enable it to be more efficient [24,25]. The respondents in this study mentioned the majority of physicians and nurses in the frontline of healthcare delivery in Burundi were not consulted before the design and implementation of the performance-based incentive scheme. This means they were unable to offer their inputs on how the incentive schemes should have been designed and on what kind of indicators should have been taken into consideration.

### Embedding of PBF in the overall strategy of quality improvement

The findings of this study reveal that performance-based incentives alone cannot help healthcare providers attain wholesale improvement in quality of care. Furthermore, the overall context of each health setting, along with the interaction of PBF with other quality improvement strategies, should be taken into consideration when designing PBF schemes. Based on the findings of this study, five major challenges may hinder PBF from stimulating high-quality care. Firstly, general staffing levels are a fundamental issue in most facilities with Burundi in particular having a severe lack of both physicians and specialists. In Burundi, all hospitals and 93% of health centres do not meet the minimum staffing requirements [39]. The World Health Organization reported in 2010 that Burundi had one physician per 20,000 people and two nurses and midwives per 10,000 people [54]. Secondly, apart from the staffing levels, comprehensive improvement in healthcare infrastructure and management is a wide-ranging issue, embracing facilities and their equipment, administration, and organizational structure. This issue could be coupled with a general lack of utility infrastructure such as the provision of sufficient clean water and electricity. Thirdly, the logistics and management of medical supplies procurement remain a challenge. In Burundi, there is a central, government-owned medical store, operating as a monopoly. It is questionable whether it has the capacity to distribute medical supplies in an appropriate manner to all health facilities. Fourthly, good knowledge and appropriate skills, and improvements in education and training for health workers are fundamental requirements in the provision of healthcare. In addition, access to health services through different types of health insurance and social policies is a further challenge to these communities. With such an extensive range of basic healthcare structural issues to be addressed in Burundi, as well as in other developing countries, the PBF schemes are used to developing the standard of healthcare delivery in these areas. Many PBF schemes include health facility administration in incentivized indicators to foster the administration and management of health facilities [2]. However, giving an incentive to only administrative indicators may not bring about big changes in healthcare provision improvement if the knowledge and skills in facility management of health administrators are not upgraded. Fritsche and colleagues mentioned in their PBF ‘toolkit’ that health facility autonomy and governance should be improved to enable health facilities to make their own specific healthcare improvement plans [2].

**Figure 2.**
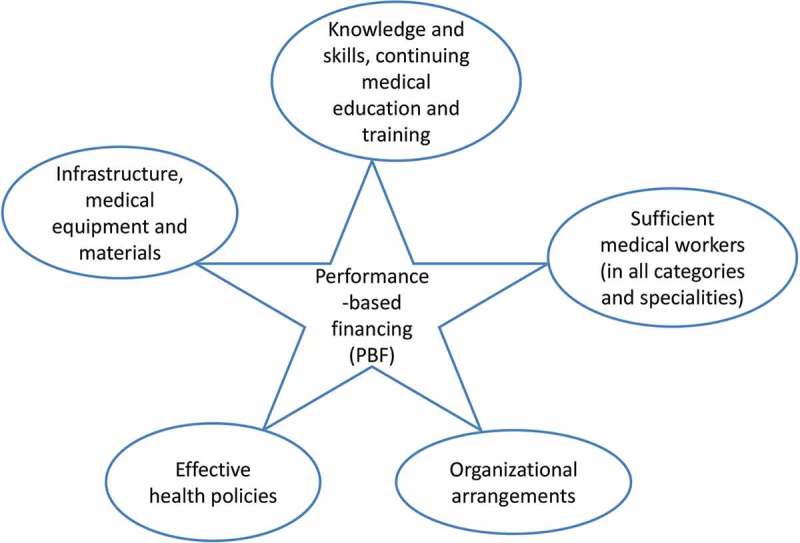
The core health strategies that interact with PBF for improving healthcare quality.

Beyond the financial incentive effect, PBF is being utilized as a ‘quality-centred’ strategy and as a feedback tool, playing a crucial role in showing where shortcomings in quality of care exist and ‘pushing’ health policy-makers to address them. The findings of this study show that the hospitals invested part of the additional revenues in quality improvements such as in medical staff and equipment, drugs, and infrastructure. A study by Meessen and Sekabaraga suggests that PBF may address several structural problems in the healthcare delivery systems in developing countries [55]. As discussed, Figure 2 highlights some important health strategies that should positively interact with PBF schemes, avoiding the challenges in implementing the schemes and thereby achieving the desired high quality of care.

## Challenges in implementing PBF schemes

PBF schemes can face different challenges in the improvements they seek to attain. Firstly, if the incentives are too small, or there is a delay in staff remuneration, the improvement achieved may start to wane as the staff become frustrated and reduce their efforts. The second challenge, as previously touched upon, is the poor infrastructure and the scarcity of qualified staff. Previous studies confirm these observations showing how health facilities, with good functional infrastructure and personnel, demonstrate a better capacity to respond well to PBF schemes than those with little investment and poor health facility management [2,10]. Thirdly, evidence from this study shows that the lack of enough medical personnel and the high workload caused by the desire to pursue incentivized quality indicators could put undue physical and emotional pressures on already overstretched health workers. The shortage of general and specialist physicians risks a poor quality of care if the additional administrative workload of achieving incentives distracts from care. On the other hand, interviewees stated that the efficiency in filling out patient medical records helped to improve the documentation of patient clinical information, resulting in better patient referral. The increase of patients and the high workload caused by the shortage of medical personnel may explain why the health personnel complained about the time spent in filling out patient records. And this may lead to ‘gaming’ the PBF system by reporting services that were not provided as this was sometimes the case in our study settings. Nonetheless, the filling out of patient medical records in an appropriate manner is of paramount importance for high-quality care and for patient safety. Sufficient medical documentation is very important for further treatments and treatment follow-ups, and eases the reference and counter-reference systems. PBF supports making comprehensive patient medical information available.

Finally, linking free health services to performance-based bonuses may create adverse effects on the quality of these services and on the income of health facilities if the unit bonuses are lower than the marginal costs of providing these services. For example, in Burundi, the unit bonus for a caesarean section is US$ 20 while its costs are estimated at US$ 130 [38]. A study by Nimpagaritse and Bertone showed that the free maternal and child health services in Burundi led to a reduction of financial flows, to drug stock-outs, to a reduction of the quality of care, and to a decrease of investments in hospitals due to lack of preparation in implementing the policy [56].

In relation to the aforementioned challenges, a study in different developing countries found out that weak systems of healthcare provision, inadequate training of medical staff, inadequate staffing, inadequate reporting and exchange of information, unavailable medical equipment and supplies, among others, result in poor provision of healthcare [57]. Anecdotal evidence indicates that performance-based incentives are here to stay [58,59], so the aforementioned challenges have to be appropriately addressed to enable PBF to achieve the desired effects.

## Conclusions

The findings of this study show that PBF motivated and positively influenced physicians and nurses to improve the healthcare provision in the studied health facilities. The most significant levels of improvement were attained in healthcare utilization, and in the structural and process measures of the quality of care. The respondents could not testify to improvement in health outcomes. Further research about the effect of PBF on health outcomes is much needed. This study suffered time and financial constraints which resulted in a very modest interview sample in the small geographical location of one province. The statements may not represent the views of all health workers at national level. However, as mentioned elsewhere, the province of Gitega is one of the three provinces (Bubanza, Cankuzo, and Gitega) that piloted PBF at its first implementation in Burundi in 2006. The health workers in Gitega Province had 5 years of experience with PBF by the time of this study. The triangulation of interview data with evidence from the literature should have outweighed the problems related to the small sample size. Notwithstanding these limitations, the findings of this study are relevant to the stakeholders of PBF programmes and are useful for health policy-makers. The study flags two main issues about the challenges of defining a satisfactory bonus level for increasing the satisfaction and the performance of medical workers. Firstly, it would appear that the size of performance incentives for health workers strongly varied among health facilities, and this may create staff frustrations in health facilities with small incentives. Secondly, a conflict of interests between the hospital management and the frontline practitioners would seem to be evident when staff need the basic financial incentives to top up their low salaries, while the hospital management is keen to invest in quality improvements and equipping the facility, often at the expense of those delivering the care in that facility. It could, however, be argued that somewhat of a ‘chicken and egg’ situation is in play because how can good care be offered without a good facility, but how can the good facility offer good care without suitably rewarding staff for their increased efforts? The findings of this study also show how the interaction of the PBF scheme with other classical health policies (see Figure 2) is very crucial in achieving comprehensive quality improvement of healthcare provision. All things being equal, PBF schemes seem to be holistic programmes that have a strong potential to influence the improvement of healthcare delivery, especially in health settings with weak healthcare provision.

## Paper context

Performance-based financing (PBF) has motivated and influenced the health providers in low-resource settings of Burundi to improve the structure and process measures of healthcare. However, the small and often delayed PBF bonuses may hamper the long-term effectiveness and sustainability of PBF. The views of frontline health workers should be taken into consideration for designing effective PBF schemes. A positive interaction of PBF schemes with other health policies is desired for improving the whole healthcare delivery system.

## References

[CIT0001] Gorter AC, Ir P, Meessen B. (2013). Evidence review, results-based financing of maternal and newborn health care in low- and lower- middle-income countries, study commissioned and funded by the German federal ministry for economic cooperation and development (BMZ) through the sector project PROFILE at GIZ – German society for international cooperation.

[CIT0002] Fritsche GB, Soeters R, Meessen B (2014). Performance-based financing. Toolkit.

[CIT0003] Eijkenaar F, Emmert M, Scheppach M (2013). Effects of pay-for-performance in health care: a systematic review of systematic reviews. Health Policy.

[CIT0004] Soeters R (2014). PBF in action. Theory and instruments. PBF Course Guide.

[CIT0005] Cromwell J, Trisolini MG, Pope GC (2011). Pay for performance in Healthcare: Methods and Approaches. RTI Press Publication. NO. BK- 002-1103.

[CIT0006] Doran T, Kontopantelis E, Valderas JM (2011). Effect of financial incentives on incentivised and non-incentivised clinical activities: longitudinal analysis of data from the UK quality and outcomes framework. BMJ.

[CIT0007] Cannon MF (2007). Pay –for- performance: is medicare a good candidate?. Yale J Health Policy Law Ethics.

[CIT0008] Bufalino V, Peterson ED, Burke GL (2006). Payment for quality: guiding principles and recommendations. principles and recommendations from the American heart association’s reimbursement, coverage, and access policy development workgroup. Circulation.

[CIT0009] Doran T, Fullword C, Gravelle H (2006). Pay-for- Performance programs in family practices in the United Kingdoms. N Eng J Med.

[CIT0010] Werner RM, Kolstad JT, Stuart EA (2011). The effect of pay-for-performance in hospitals: lessons for quality improvement. Health Aff.

[CIT0011] Travis P, Bennett S, Haines A (2004). Overcoming health systems constraints to achieve the Millennium development goals. Lancet.

[CIT0012] Fryatt R, Mills A, Nordstrom A (2010). Financing of health systems to achieve the health Millennium development goals in low-income countries. Lancet.

[CIT0013] Dieleman M, Toonen J, Touré H (2006). The match between motivation and performance management of health sector workers in Mali. Hum Resour Health.

[CIT0014] Dieleman M, Harnmeijer JW (2006). Improving health worker performance: in search of promising practices.

[CIT0015] Rowe AK, De Savigny D, Lanata CF (2005). How can we achieve and maintain high-quality performance of health workers in low-resource settings?. Lancet.

[CIT0016] Soeters R, Vroeg P (2011). Why there is so much enthusiasm for performance-based financing, particularly in developing countries. Bull World Health Organ.

[CIT0017] Berenson RA, Pronovost PJ, Krumholz HM (2013). Achieving the potential of health care performance measures: timely analysis of immediate health policy issues.

[CIT0018] Eijkenaar F (2013). Key issues in the design of pay for performance programs. Eur J Health Econ.

[CIT0019] Eijkenaar F (2012). Pay-for-performance in health care: an international overview of the initiatives. Med Care Res Rev.

[CIT0020] Robinson JC (2001). Theory and practice in the design of physician payment incentives. Journal of Public Health and Health Care Policy.

[CIT0021] CMS (Centers for Medicare and Medicaid Services) (2011). Roadmap for Implementing Value Driven Healthcare in the Traditional Medicare Fee-for-Service Program.

[CIT0022] Basinga P, Gertler PJ, Binagwaho A (2011). Effect on maternal and child health services in Rwanda of payment to primary healthcare providers for performance: an impact evaluation. Lancet.

[CIT0023] Ryan AM, Blustein J (2011). The effect of the masshealth hospital pay-for-perfomance programm on quality. Health Serv Res.

[CIT0024] Edwards N (2005). Doctors and managers: building a new relationship. Clin Med.

[CIT0025] Edwards N (2003). Doctors and managers: poor relationships may be damaging patients – what can be done?. Qual Saf Health Care.

[CIT0026] Casalino LP, Alexander GC, Lei Jin L (2007). General internists’ views on pay-for-performance and public reporting of quality scores: a national survey. Health Aff.

[CIT0027] Reiter KL, Nahra TA, Jeffrey A (2006). Hospital responses to pay-for-performance incentives. Health Serv Manage Res.

[CIT0028] Locke RG, Srinivasan M (2008). Attitudes toward pay-for-performance initiatives among primary care osteopathic physicians in small groups practices. J Am Osteopath Assoc.

[CIT0029] Damberg CL, Raube K, Teleki SS (2009). Taking stock of pay-for-performance: a candid assessment from the front lines. Health Aff.

[CIT0030] Paul E, Sossouhounto N, Eclou DS (2014). Local Stakeholder´s perceptions about the introduction of performance-based financing in Benin: a case study in two health districts. Int J Health Policy Manag.

[CIT0031] Paul F. (2009). Health worker motivation and the role of performance based finance systems in Africa: a qualitative study on health worker motivation and the Rwandan performance based finance initiative in district hospitals. London School of Economics and Political Science working paper series 08-96.

[CIT0032] Bhatnagar A, George AS (2016). Motivating health workers up to a limit: partial effects of performance-based financing on working environments in Nigeria. Health Policy Plan.

[CIT0033] Khim K (2016). Are health workers motivated by income? Job motivation of Cambodian primary health workers implementing performance-based financing. Glob Health Action.

[CIT0034] Aninanya GA, Howard N, Williams JE (2016). Can performance-based incentives improve motivation of nurses and midwifes in primary facilities in northern Ghana?. Glob Health Action.

[CIT0035] Yé M, Diboulo E, Kagoné M (2016). Health workers preferences for performance-based payment schemes in a rural health district in Burkina Faso. Glob Health Action.

[CIT0036] Bertone MP, Meessen B (2013). Studying the link between institutions and health system performance: a framework and an illustration with the analysis of two performance-based financing schemes in burundi. Health Policy Plan.

[CIT0037] Busogoro J, Beith A (2010). Pay for performance for improved health in Burundi.

[CIT0038] Ministry of Health Burundi (2010). Manuel de procédures pour la mise en œuvre du financement basé sur la performance au Burundi.

[CIT0039] Ministry of Health Burundi (2013). Cartographie des Ressources humaines ein santé, Rapport.

[CIT0040] Chaumont C, Muhorane C, Moreira-Burgos I (2015). Maternal and reproductive health financing in Burundi: public-sector contribution levels and trends from 2010 to 2012. BMC Health Services Research.

[CIT0041] World Bank Health Results Innovation Trust Fund (2013). Results-based financing for health.

[CIT0042] Cordaid-Ministry of Health Burundi (2015). Resultats de l´enquête ménage et l´enquê qualité de base.

[CIT0043] Sibomana S, Reveillon M (2015). Performance based financing of priority health services. Burundi backgroup paper. WHO. Improving health system efficiency.

[CIT0044] Campbell SM, Reeves D, Kontopantelis E (2009). Effects of pay for performance on the quality of primary care in England. N Eng J Med.

[CIT0045] Soeters R (2009). PBF in action. Theory and instruments. PBF course guide.

[CIT0046] Bonfrer I, Soeters R, Van de Poel E (2014). Introduction of performance based financing in Burundi associated with improvements in care and quality. Health Aff.

[CIT0047] Rudasingwa M, Soeters R, Bossuyt M (2015). The effect of performance-based financial incentives on improving health care provision in Burundi: a controlled cohort study. Glob J Health Sci.

[CIT0048] Bryman A (2008). Social Research Methods.

[CIT0049] Clarke V, Braun V (2013). Teaching thematic analysis: Overcoming challenges and developing strategies for eﬀective learning. Psychologist.

[CIT0050] Bonfrer I, Van de Poel E, Van Doorslaer E (2014). The effects of performance incentives on the utilization and quality of maternal and child care in Burundi. Soc Sci Med.

[CIT0051] Das J, Gertler PJ (2007). Variations in practice quality in five low-income countries: a conceptual overview. Health Aff.

[CIT0052] Gertler P, Vermeersch CM (2012). Using performance incentives to improve health outcomes. Policy Res Work Pap.

[CIT0053] Leonard KL, Masatu MC (2010). Professionalism and the know-do gap: exploring intrinsic motivation among health workers in Tanzania. Health Econ.

[CIT0054] World Health Organization (WHO) (2010). World Health Statistics.

[CIT0055] Meessen B, Soucat A, Sekabaraga C (2011). Performance-based financing: just a donor fad or a catalyst towards comprehensive health-care reform?. Bull World Health Organ.

[CIT0056] Nimpagaritse M, Berton MP (2011). The sudden removal of use fees: the perspective of a frontline manager in Burundi. Health Policy Plan.

[CIT0057] Wilson RM, Michel P, Olsen S (2012). Patient safety in developing countries: retrospective estimation of scale and nature of harm to patients in hospital. BMJ.

[CIT0058] Honda A (2013). 10 best resources on … pay for performance in low- and middle-income countries. Health Policy Plan.

[CIT0059] Ryan AM, Blustein J (2012). Making the best of hospital pay for performance. N Engl J Med.

